# Zn Metal Anodes for Zn-Ion Batteries in Mild Aqueous Electrolytes: Challenges and Strategies

**DOI:** 10.3390/nano11102746

**Published:** 2021-10-17

**Authors:** Vo Pham Hoang Huy, Luong Trung Hieu, Jaehyun Hur

**Affiliations:** Department of Chemical and Biological Engineering, Gachon University, Seongnam 13120, Gyeonggi, Korea; vophamhoanghuy@yahoo.com.vn (V.P.H.H.); LuongTrungHieu290694@gmail.com (L.T.H.)

**Keywords:** Zn metal anode, aqueous Zn ion batteries, mildly acidic electrolyte, dendrite-free, hydrogen evolution reaction suppression

## Abstract

Over the past few years, rechargeable aqueous Zn-ion batteries have garnered significant interest as potential alternatives for lithium-ion batteries because of their low cost, high theoretical capacity, low redox potential, and environmentally friendliness. However, several constraints associated with Zn metal anodes, such as the growth of Zn dendrites, occurrence of side reactions, and hydrogen evolution during repeated stripping/plating processes result in poor cycling life and low Coulombic efficiency, which severely impede further advancements in this technology. Despite recent efforts and impressive breakthroughs, the origin of these fundamental obstacles remains unclear and no successful strategy that can address these issues has been developed yet to realize the practical applications of rechargeable aqueous Zn-ion batteries. In this review, we have discussed various issues associated with the use of Zn metal anodes in mildly acidic aqueous electrolytes. Various strategies, including the shielding of the Zn surface, regulating the Zn deposition behavior, creating a uniform electric field, and controlling the surface energy of Zn metal anodes to repress the growth of Zn dendrites and the occurrence of side reactions, proposed to overcome the limitations of Zn metal anodes have also been discussed. Finally, the future perspectives of Zn anodes and possible design strategies for developing highly stable Zn anodes in mildly acidic aqueous environments have been discussed.

## 1. Introduction

Renewable energy supplies have drawn worldwide attention because of the scarcity of fossil fuels and the increasing global warming [1,2,3]. Nevertheless, renewable energy resources, such as solar, wind, and geothermal are interrupted by various climatic and natural factors; thus, grid-scale energy is a vital underpinning for the continued development of large-scale energy storage techniques, and secondary batteries are an indispensable choice for achieving this [4,5,6]. Since their successful commercialization in the 1980s, lithium-ion batteries (LIBs) have dominated the energy market and have been employed in various applications, from portable electronics to grid-scale energy storage systems [7,8,9,10,11,12,13,14,15,16,17,18,19,20,21,22]. Nevertheless, the growth of the LIB technology has been limited owing to its safety issues, limited Li supplies, and high intrinsic prices [23,24,25,26]. Sodium-ion batteries (SIBs) and potassium-ion batteries (PIBs) have been intensively studied as alternatives to LIBs. However, SIBs and PIBs utilize volatile, flammable, and toxic organic electrolytes, which lead to safety and environmental issues [27,28,29,30,31,32,33]. Therefore, various efforts have been made to develop potential alternatives to these batteries that are suitable for grid-scale applications [34,35,36,37]

Aqueous batteries are very promising alternatives for LIBs, SIBs, and PIBs because aqueous electrolytes are inexpensive and environmentally friendly, and hence can alleviate the risk of fire hazards and explosions [38,39,40,41,42]. In addition, fast charging and high energy density can be achieved with aqueous batteries because water has considerably higher (by two to three orders of magnitude) ionic conductance (i.e., ~1 S cm^−1^) than most organic solvents [43,44,45].

**Figure 1:**
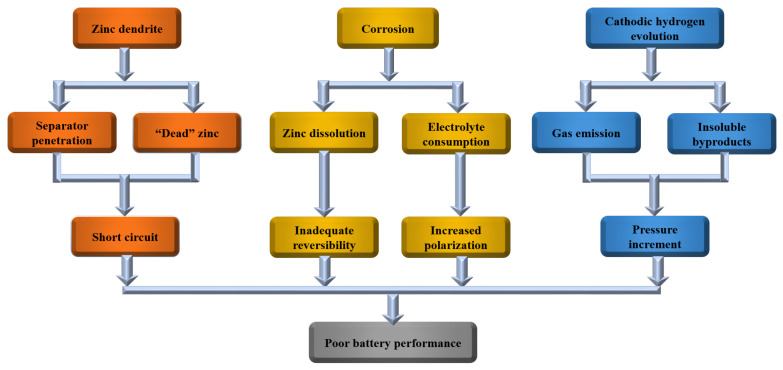
Major limitations of Zn metal anodes for battery applications.

**Figure 2:**
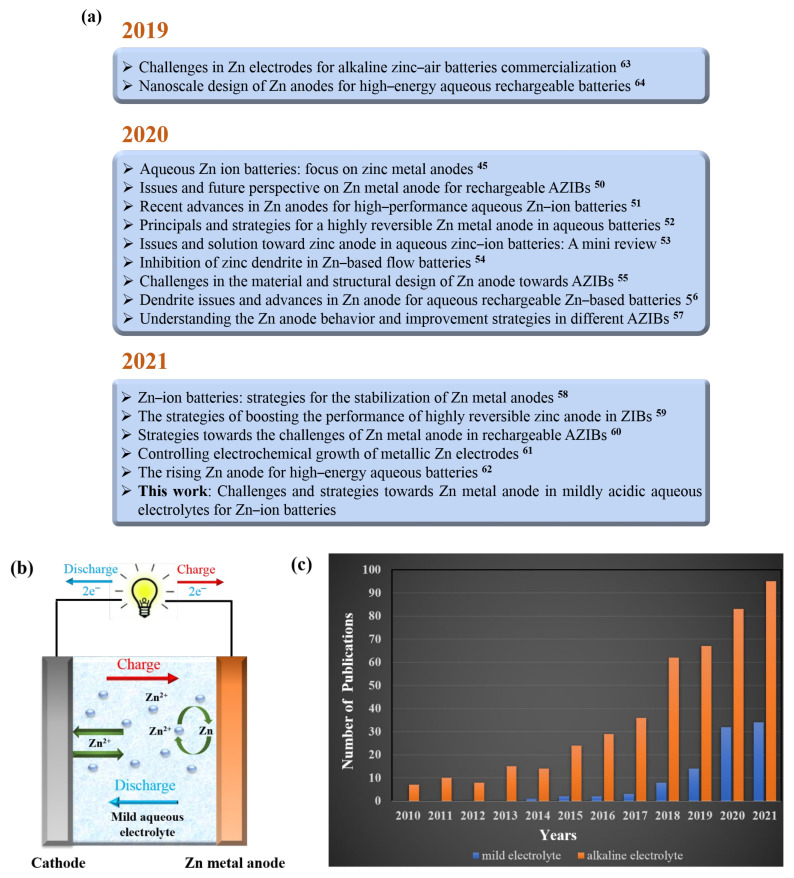
(**a**) Brief summary of recent reviews on Zn metal anodes for AZIBs [45,50,51,52,53,54,55,56,57,58,59,60,61,62,63,64], (**b**) a typical full cell battery configuration of AZIBs in a mildly acidic aqueous electrolyte. (**c**) The number of publications on AZIBs from 2010 to 2021 (search from Google Scholar; search time: 7 September 2021).

Among the various aqueous batteries investigated to date, aqueous Zn ion batteries (AZIBs) have recently emerged as a promising technology capable of satisfying today’s stringent battery requirements. The use of Zn metal as an anode in aqueous media is a unique feature of these batteries that contributes to their excellent performance. First, as compared to monovalent metals, such as Li or Na, Zn is not only readily available and cheap but is also known for its non-toxicity and chemical durability in aqueous systems [46]. Second, in mildly acidic electrolytes (pH = 4–6), Zn oxidizes to Zn^2+^ without producing intermediate products and possesses a high overpotential for the hydrogen evolution reaction (HER) [47]. Third, given the relatively narrow operating window in which gas (H_2_ and O_2_) formation can be prevented in water, Zn has a redox potential of −0.76 V (vs. the typical hydrogen electrode), which is suitable for battery applications. Finally, in its metallic state, Zn has a high theoretical capacity (820 mAh g^−1^, 5854 mAh L^−1^) [45,48,49]. However, the practical applications of AZIBs are significantly hindered by the formation of Zn dendrites and the occurrence of side reactions (HER and corrosion) on the Zn anode. Zn dendrites can transpierce the battery separator, causing a short circuit, while the corrosion of the Zn metal anode results in irreversible electrolyte consumption and the generation of insoluble byproducts, increasing the electrode polarity and degrading the battery performance. On the other hand, the H_2_ gas produced from the electrochemical reaction elevates the internal pressure of the battery, causing safety hazards, as illustrated in Figure 1. As a result, in recent years, various efforts have been made to develop appropriate strategies to overcome the limitations associated with Zn metal anodes. Figure 2a highlights some representative reviews on Zn metal anodes published over the last three years [45,50,51,52,53,54,55,56,57,58,59,60,61,62,63,64]. Currently, AZIBs can be largely categorized into two types: those with alkaline electrolytes, normally Zn-Ag, Zn-air, and Zn-Ni batteries [65,66,67]. and those with mildly acidic electrolytes, such as Zn-MnO_2_ and Zn-V_2_O_5_ batteries (as shown in Figure 2b) [68,69]. Although Zn-based batteries with alkaline electrolytes generally have higher energy densities than those with non-alkaline electrolytes, mildly acidic aqueous electrolytes can effectively inhibit the growth of Zn dendrites, thus improving the cyclic performance of the battery through highly reversible electrochemical plating/stripping of Zn^2+^ on the Zn anode. As shown in Figure 2c, the number of publications on aqueous electrolytes with mildly low pH has increased significantly year by year. However, comprehensive and systematic reviews on the challenges and strategies for the development of next-generation Zn metal anodes suitable for application in mildly acidic electrolytes are scarce. Therefore, it will be beneficial to review the recent studies on Zn metal anodes in mildly acidic electrolytes to provide guidelines for the development of high-performance AZIBs.

This review summarizes the strategies for the development of high-performance AZIB anode materials. First, the major limitations of zinc metal anodes are discussed followed by the approaches developed to overcome them. Finally, the future perspectives for next-generation AZIB research are discussed. This review will be beneficial for the rational design of Zn metal anodes in mildly acidic aqueous systems.

## 2. Challenges in the Commercialization of Zn Metal Anodes

### Zn Anode Reactions

**Figure 3:**
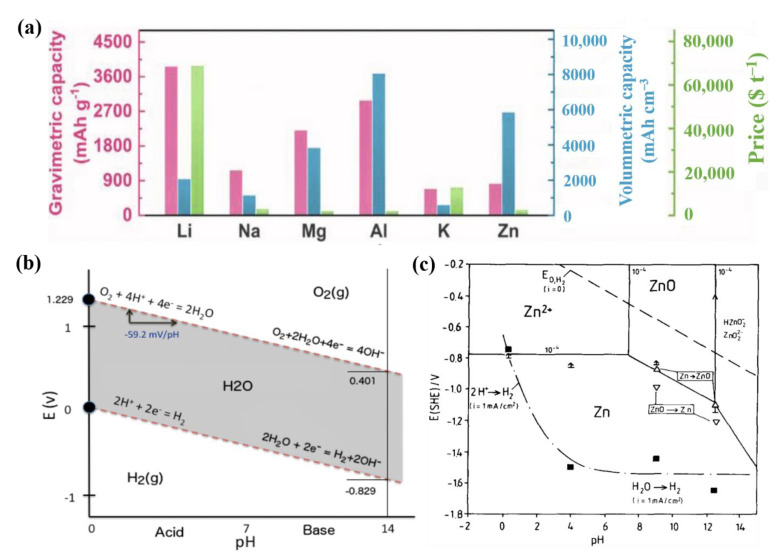
(**a**) Gravimetric capacity, volumetric capacity, and price of typical metal anodes. Reprinted with permission from Liang et al. [58]. Copyright 2021 Wiley-VCH GmbH. (**b**) Pourbaix diagram of water. Reprinted with permission from Zeng et al. [75]. Copyright 2019 Elsevier B.V. (**c**) Pourbaix diagram of the Zn/H_2_O system with HER overpotential considerations. Reprinted with permission from Wippermann et al. [76]. Copyright 1990 Elsevier Ltd.

To date, various types of metal-ion aqueous rechargeable batteries (M = Li, K, Na, Ca, Zn, Mg, Al) operable in the pH range of 3–11 have been extensively investigated [70,71,72]. While most metals do not function properly in aqueous media because of their limited redox voltages, beyond which water becomes unstable, Zn exhibits advantages, such as a low redox potential (−0.76 V vs. the standard H_2_ evolution), high overpotential for the HER, and high theoretical capacity (820 mAh g^−1^, 5855 mAh cm^3^), which make it suitable for application as a metal anode in aqueous systems [57,73,74] (Figure 3a–c).

In AZIBs, the cathode reactions depend on the cathode material, whereas the Zn anode reactions are highly influenced by various electrolyte conditions. In alkaline media, Zn metal anodes oxidize to the zincate ion complex (Zn(OH)_4_^2−^) because of the presence of numerous OH^−^ ions in the surroundings [77]. When the concentration gradient changes, these zincate ions disperse away from the electrode surface, causing the loss of the active material. In addition, when the solubility of the zincate ions decreases locally, a passive ZnO layer is formed on the electrode surface [78]. This precipitate results in dendritic growth and/or passivation, which reduces the rechargeable capacity of the alkaline aqueous electrolyte battery.

In mildly acidic aqueous media (virtually neutral media), the charge carrier is mainly Zn^2+^ owing to the lack of OH^−^. Unlike the case in alkaline systems, only Zn^2+^ ions are reversibly stripped/plated on the Zn metal anode surface during the battery operation in mildly acidic aqueous media, which is similar to the Li metal anode mechanism in LIBs. Despite this, Zn electrodes are highly reactive and induce many side reactions with the electrolyte, which results in low Coulombic efficiency (CE) and poor cycle life. Typically, highly reversible Zn plating/stripping is possible in mildly acidic aqueous electrolytes, such as ZnSO_4_ or Zn(CF_3_SO_3_)_2_. In these media, the reactions of the Zn metal anode occur as follows: 

Discharge process:

Zn → Zn^2+^ + 2e^−^ (Zn stripping) (1)

Charge process:

Zn^2+^ + 2e^−^ → Zn (Zn plating) (2)

**Figure 4:**
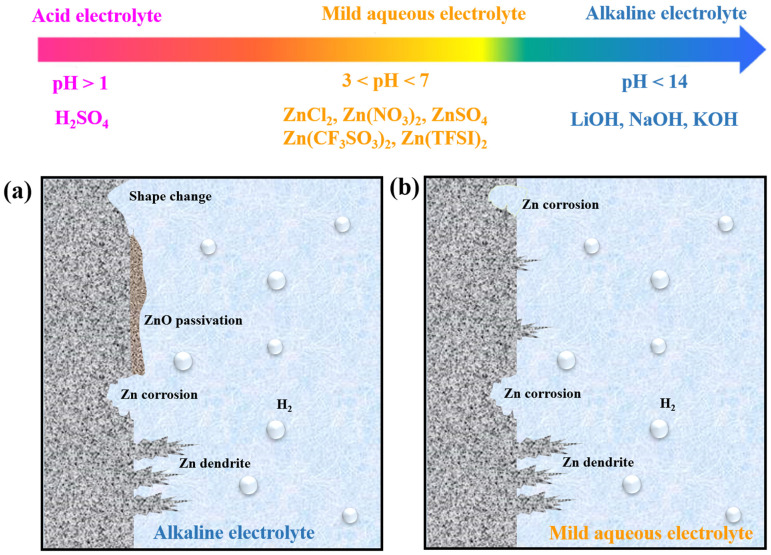
Schematic illustration of the phenomena observed on Zn electrodes in (**a**) alkaline and (**b**) mild aqueous electrolytes.

Figure 4 summarizes the common problems encountered in alkaline (Figure 4a) and mild-pH media (Figure 4b). Although some typical problems (e.g., shape change and ZnO passivation) encountered in alkaline electrolytes are not serious in mildly acidic electrolytes because of their different Zn electrode reaction mechanisms, the most common problems, such as dendrite growth and H_2_ evolution are the main reasons for the occurrence of irreversible reactions in ZIBs, which reduces the CE of Zn electrodes and deteriorates their performance. In the following section, we will focus on these issues of Zn metal anodes in mildly acidic electrolyte systems.

## 3. Assembly and Test Technology of Zn-Ion Batteries with Zn Metal Anodes

### 3.1. Cell Assembly

**Figure 5:**
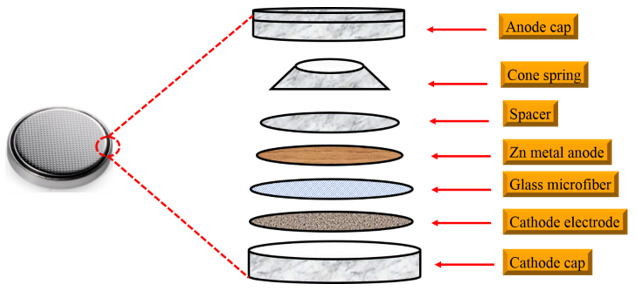
Components of the AZIB in coin cell assembly.

A typical AZIB consists of the following components: Zn metal (anode), an aqueous electrolyte, a separator, and cathode material. Figure 5 and Table 1 show the list of components and typical materials used in the AZIB coin-cell. Because of its tunnel or layered structure which allows reversible insertion/extraction of Zn^2+^ ions, MnO_2_ has been extensively used as a cathode material in the early stages of mild aqueous ZIBs. Furthermore, manganese (Mn)-based oxides have been considered as promising energy storage materials due to their low cost, abundance, environmental friendliness, low toxicity, and numerous valence states (Mn^0^, Mn^2+^, Mn^3+^, Mn^4+^, and Mn^7+^) [79,80]. The cathode material is prepared as a slurry by mixing it with conductive carbon and polymer binder and dispersing it in the organic solvent. The slurry is cast on the current collector (typically, stainless steel) with a well-defined thickness using the doctor blade technique. The electrolyte, as a component in direct contact with the Zn anode and directing the plating/stripping process of Zn, is critical to the electrochemical reversibility and stability of the Zn metal anode in AZIB systems. Currently, the ZnSO_4_ or Zn(CF_3_SO_3_)_2_ salt-based electrolytes are considered to be promising electrolytes in mild AZIBs [57]. Although a commercial Zn foil has been widely adopted as an anode and directly used as a current collector in most ZIBs, electrodeposited Zn electrodes on appropriate current collectors can also be the Zn metal anodes. The selection of the current collector is of great importance for the deposited Zn electrode; carbon-based, copper-based, and MOF-based current collectors have been widely used to support Zn, owing to their great chemical and electrochemical stability in various electrolytes, robust mechanical strength to accommodate deposition, high electrical conductivity, and close affinity for Zn [81]. 

### 3.2. Cell Test

**Figure 6:**
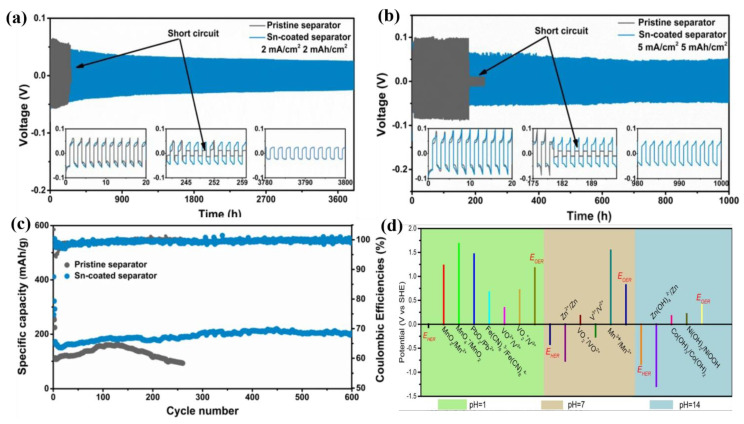
Cycling performance of Zn/Zn symmetric cells tested at (**a**) 2 mA cm^−2^ and 2 mAh cm^−2^, (**b**) 5 mA cm^−2^ and 5 mAh cm^−2^, (**c**) cycling performance at 0.3 A g^−1^. Reprinted with permission from Zhen et al. [83]. (**d**) The standard potentials of redox couple in some reported cathode materials in ZIB systems. Reprinted with permission from [84]. Copyright 2021 Wiley-VCH GmbH.

In AZIBs, the cell tests are typically performed with symmetric and full cell configuration. In evaluating the symmetrical cell performance, two identical electrodes (Zn/Zn) are used to investigate the coulombic efficiency (CE) and degree of polarization as a function of cycle number. CE is one of the important parameters to quantify the reversibility of an electrochemical system. The tendency of CE can be a useful tool in accurately predicting cycle life and the reversibility of Zn deposition/stripping, which is calculated based on the capacity ratio of stripping to plating. In Zn/Zn symmetric cells, there are three key parameters: cycle life, current density, and cycling capacity, which can provide a comprehensive picture of the electrochemical performance of symmetric batteries. Figure 6 is an example of evaluating a material’s effectiveness through the symmetric cell test [83]. The stripping/plating stability and polarization of electrodes were evaluated at various current densities and cycling capacities with the symmetric cell test. For example, Yu et al. demonstrated the enhanced cycling stability of a Zn metal anode by using a Sn-coated separator. It was shown that a Zn/Zn symmetric cell with Sn-coated separator exhibits a dramatically improved cycle life of 3800 h (current density: 2 mA cm^−2^, cycling capacity: 2 mAh cm^−2^), 1000 h (current density: 5 mA cm^−2^, cycling capacity: 5 mAh cm^−2^) (Figure 6a,b). Furthermore, a highly reversible stripping/plating reversibility was achieved with a CE of ~99% at 0.3 A g^−1^ after 600 cycles for the Sn-coated separator (Figure 6c). 

In a full cell measurement, two different materials are used for cathode and anode which determines the voltage range that can be applied. In most cases, the practical output voltage of AZIBs cannot be fully in agreement with the theoretical voltage range. Theoretically, the maximum output voltage can be easily obtained by choosing a strong oxidizer for the cathode and a strong reducing agent for the anode, respectively. However, for AZIBs with Zn metal anode, the electrode potential is fixed (−0.76 V vs. SHE in a neutral or acidic solution, 1.23 V vs. SHE in an alkaline solution). Thus, the key to constructing high-voltage AZBs is to choose a suitable cathode material that has a lower electrode potential than Zn metal [84]. Figure 6d shows the standard potentials of redox couples in some reported cathode materials. For instance, Zn//MnO_2_ batteries in a mild electrolyte deliver an output voltage that is below 1.5 V (vs. Zn/Zn^2+^). The practical energy density, power density, and energy efficiency of the cell can be estimated from the full cell configuration.

## 4. Drawbacks of Zn Metal Anodes in Mildly Acidic Electrolytes

### 4.1. Zn Dendrite Growth

**Figure 7:**
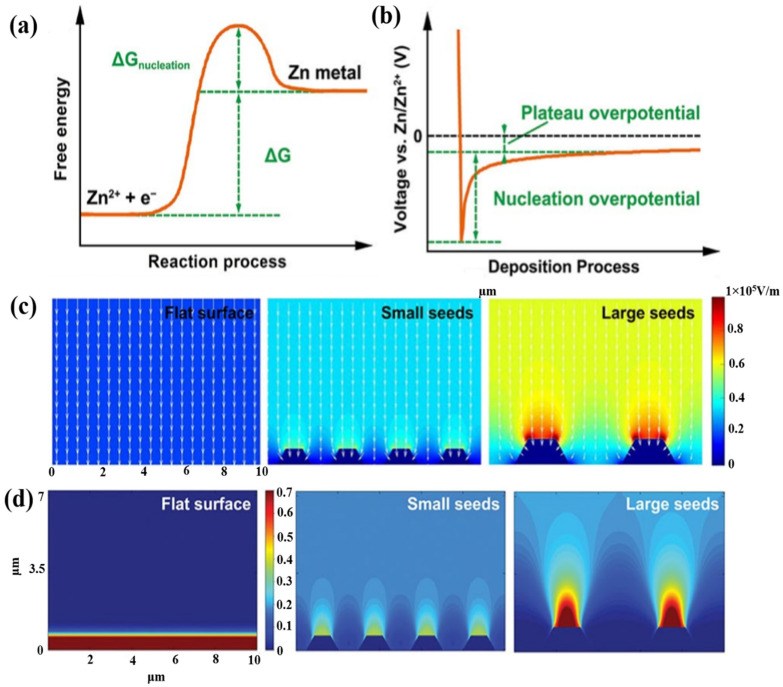
(**a**) Energy barrier for the Zn nucleation process, (**b**) voltage profile during Zn deposition. Reprinted with permission from Pei et al. [87]. Copyright 2017 American Chemical Society. Simulation of (**c**) electric field and (**d**) ion distribution on the Zn anode surface under different dendrite formation conditions: flat surface, small dendritic seeds, and large dendritic seeds. Reprinted with permission from [90]. Copyright 2019 WILEY-VCH Verlag GmbH&Co. KgaA, Weinheim.

In AZIBs with mild aqueous electrolytes, the formation and growth of Zn dendrites within the battery is a major issue. The term “Zn dendrite” refers to a wide range of Zn morphologies with sharp ends or edges that pierce the separator and eventually lead to a short circuit and the breakdown of the cell. When Zn dendrites detach from an electrode, “dead” or “orphaned” Zn is easily generated, leading to a quick drop in the CE of the battery and an irreversible drop in its capacity. The Zn^2+^ concentration gradient in the proximity of the electrode surface influences the formation of Zn dendrites in a mildly acidic electrolyte. Zn ions diffuse on the electrode surface and accumulate easily on the nucleation sites, forming an initial protrusion. This further exacerbates the unequal electric field distribution, causing more Zn ions to be collected and the growth of dendrites over time via repeated plating/stripping at the anode. This inhomogeneous Zn^2+^ deposition directly affects the development of Zn dendrites [53,85]. Figure 7a shows the free energy diagram of the Zn reduction process, where Zn ions must overcome a nucleation energy barrier to reach a new solid stage. After the nucleation, Zn tends to be plated and accumulated in the pre-deposited region owing to the combined actions of the electric field and concentration gradient [86,87]. Figure 7b shows the voltage profile during the Zn deposition process. The difference between the potential minima and the subsequent stable potential, which indicates the thermodynamic cost of establishing a crucial atom cluster, is known as the nucleation overpotential, whereas the plateau overpotential is related to the Zn growth after the initial nucleation [88,89]. The lower the value of these two parameters, the better is the progress of the nucleation and growth processes (with low energy consumption). In addition, the electric field, ion concentration, and surface energy all influence the Zn nucleation. The “tip effect” causes Zn^2+^ to be concentrated in the protruded regions with high surface energy because the electric field at these points is substantially higher than those in the remaining areas. As a result, the Zn nucleation and growth are inclined to occur at such points. The “tip effect” leads to an uneven distribution of the electric field intensity at the surface of the electrode, a non-uniform ion concentration distribution, and the preferential deposition of Zn at locations with higher Zn^2+^ concentrations (Figure 7c,d). It is obvious that the Zn^2+^ concentration is high in a region with a high electric field where dendrites are easily formed [90]. While Zn dendrites are homogeneously formed at surfaces with high surface energies, which can minimize the nucleation barriers by generating a large number of nucleation sites, irregular Zn dendrites are formed at the surfaces with a large number of nucleation barriers because of the unequal distribution of the electric field or a small number of nucleation sites [53,58,91].

**Figure 8:**
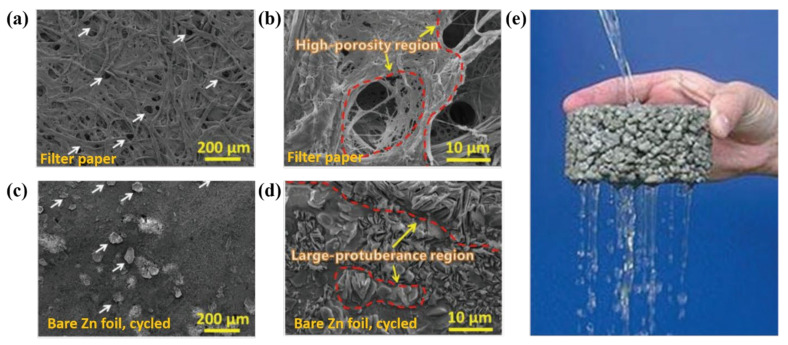
SEM images of (**a**,**b**) a fresh filter paper, and (**c**,**d**) a cycled bare Zn foil. The pores in filter papers act as “highways” for electrolyte transport, like the (**e**) pores in water-permeable bricks. Reprinted with permission from [92]. Copyright 2018 WILEY-VCH Verlag GmbH&Co. KgaA, Weinheim.

Kang et al. proposed an intriguing concept concerning the dimensions and distribution of pores in the coating material, which influences the evolution of Zn protrusions/dendrites, to understand the formation of Zn dendrites [92]. They proposed that the dimensions and dispensation of pores in a filter paper resemble those of the immense protuberances on cycled Zn foils (Figure 8a,b vs. Figure 8c,d). Therefore, a study with a filter paper can provide an understanding of the stripping/plating behavior of Zn^2+^ on Zn metal anodes. During cycling, the pores in a filter paper (an example of a porous coating material) behave similarly to the holes in water-permeable bricks (Figure 8e), forming a region highly active for Zn stripping/plating. As the number of cycles increases, additional Zn accumulates on the Zn metal surface, which makes contact with the porous regions in the coating material and generates a large number of protrusions, affecting the efficiency of the Zn^2+^ stripping/plating processes in diverse ways. First, the separator with uneven voids (such as filter paper) kinetically deteriorates the electrochemical performance of Zn anodes by limiting the local electrolyte transport. Second, the effect of the composition of the filter paper pores on the Zn dendrite evolution is pore dimension dependent; that is, a tiny pore promotes the development of small protrusions/dendrites. As a result, the dimensional characteristics of the pores in the covering material are important for regulating the Zn dendrite formation.

### 4.2. Zn Electrode Corrosion

Owing to its amphoteric nature, Zn can react with OH^−^ and H^+^ ions. The presence of a considerable number of OH^−^ species in alkaline electrolytes deteriorates the performance of Zn anodes by forming Zn(OH)_4_^2−^ or ZnO by-products. On the other hand, electrolytes with mildly low pH can limit the occurrence of such unfavorable reactions because of the presence of modest amounts of H^+^ species [93]. According to the Pourbaix diagram, although Zn metal is thermodynamically stable over a wide pH range in mild aqueous media, the corrosion caused by free water should still be considered [94]. Unlike the case of Li metal batteries, in which the solid electrolyte interface (SEI) prevents the excessive consumption of Li metal, in AZIBs, Zn deposition is hampered by H_2_ evolution, leading to severe Zn metal corrosion. When the corrosion process continues, the Zn anode surface becomes more uneven and unsafe because of the increased pressure inside the cell. A recent study demonstrated that some Zn salts (e.g., Zn(NO_3_)_2_ and Zn(ClO_4_)_2_) may play a negative role in the Zn corrosion reaction [95]. For example, in the early stages of corrosion in a neutral electrolyte, a strong oxidizing agent with NO_3_^−^ anions leads to the corrosion of the Zn foil and cathode materials. Although Zn(ClO_4_)_2_ with ClO_4_^2−^ having four O atoms and one Cl atom located at the corners and center of the tetrahedral structure, respectively, shows low reactivity, it requires an excessive operational voltage, which leads to slow reaction kinetics because of the formation of by-products [96]. Zinc halides (e.g., ZnCl_2_ and ZnF_2_) have been widely studied as Zn-based electrolytes owing to their poor oxidative properties [97]. However, there are certain disadvantages of these electrolytes: (i) the utilization of the ZnF_2_ electrolyte is limited by its low water solubility; (ii) although ZnCl_2_ is highly soluble in water, it has a narrow stable potential window for the occurrence of anodic electrochemical reactions without any side reactions, which limits its application in AZIBs. On the other hand, ZnSO_4_ has been extensively employed as an electrolyte for AZIBs because of its low cost, good solubility, wide potential window, and mild pH in water. Furthermore, in such mild ZnSO_4_ electrolytes, Zn anodes exhibit excellent dissolution/deposition reaction dynamics, little dendritic development, and moderate corrosion [98,99,100]. Nevertheless, the exact electrochemical reaction mechanism of Zn metal anodes in ZnSO_4_ electrolytes is not known yet, partly because of the formation of Zn_4_(OH)_6_SO_4_nH_2_O (ZHS). It is believed that the pH fluctuations of the electrolytes throughout the discharge process are normally regulated by ZHS according to the following reaction.

4 Zn^2+^ + SO_4_^2−^ + 6 OH^−^ + 5 H_2_O → Zn_4_(OH)_6_(SO_4_)·5 H_2_O (3)

These by-products are formed by prolonged electrolysis and Zn ion consumption, which generally reduces the plating/stripping CE of the Zn anode to some extent [13]. As a result, extra Zn is required to ensure continuous cycling to prevent the Zn anode from reaching its maximum theoretical capability. Furthermore, the inert by-products deposited on the Zn surface obstruct the ion transfer and reduce the reversibility of the Zn anode.

**Figure 9:**
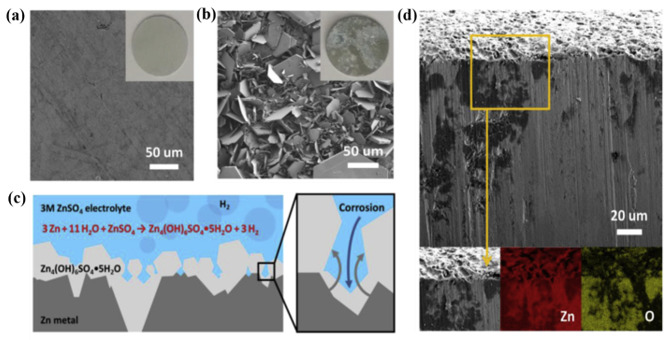
SEM images of a Zn electrode (**a**) before and (**b**) after 30 days of operation, (**c**) Chemical corrosion of the Zn metal electrode in a ZnSO_4_ electrolyte. (**d**) cross-section SEM and EDS elemental mapping results of the electrode operated for 30 days. Reprinted with permission from Cai et al. [101]. Copyright 2020 Elsevier B.V.

Recently, Cai et al. studied the corrosion process of Zn metal anodes in ZnSO_4_ electrolysis [101]. They found that the corrosion of the Zn metal anode started from the surface layer, leading to the formation of Zn_4_(OH)_6_SO_4_, followed by H_2_ evolution. Subsequently, Zn_4_(OH)_6_SO_4_ is further hydrated and converted into Zn_4_(OH)_6_SO_4_·5H_2_O, leading to the considerable corrosion of the Zn metal anode (Figure 9a–c). In this way, the Zn metal surface could not be passivated, and the corrosion proceeded until the liquid electrolyte or active Zn metal was completely consumed. Notably, the corrosion process continued until an uneven corrosion depth of 132.2 μm was achieved (Figure 9d), which could be clearly detected by the O atom signal in the energy-dispersive X-ray spectroscopy (EDS) elemental mapping image.

### 4.3. Hydrogen Evolution

Metal corrosion is a common side effect of the HER, which is another key issue limiting the applications of ZIBs. Owing to its lower electronegativity than that of H, Zn prefers to react with water in neutral or slightly acidic electrolytes [102]. In addition, the Zn^2+^ deposited on the surface of highly reactive Zn metal anodes enhances the occurrence of side reactions. The hydrogen evolution that occurs over time not only dries out the electrolyte but also speeds up the hydration of the Zn cations in the aqueous electrolyte, resulting in suboptimal Zn usage as compared to the theoretical capacity. This generates H_2_ gas, which also corrodes the Zn metal surface, deteriorates the battery performance, and poses a safety risk. Furthermore, the consumption of H^+^ in water leads to an increase in the OH^−^ ion concentration at the interface of the Zn anode/electrolyte, which produces insulating by-products and causes non-uniform Zn plating [103]. In mildly acidic electrolysis, the occurrence of the HER is inevitable because the standard reduction potential of Zn/Zn^2+^ lags behind the H_2_ evolution potential (0 V vs. SHE) [104]. as follows [105]:

Zn^2+^ + 2e^−^ ↔ Zn (−0.76 V) (4)

2 H^+^ + 2e^−^ ↔ H_2_ (0 V) (5)

Because Zn has a large overpotential for H_2_ evolution in aqueous electrolytes, the HER is not as problematic as the values indicate [106], which could be described by the Tafel equation as follows [107].

η = b logi + a(6)

where η is the H_2_ evolution overpotential, i is the current density, b is the Tafel slope constant, and a is the overpotential when the current density i is equal to the unit current density. Because b is roughly the same for all metals (0.112 V), the value of the overpotential for H_2_ evolution is mostly determined by the value of a [108]. Zn has a strong H_2_ overpotential as well as a high a value. According to the Pourbaix diagram of Zn in aqueous media, the high overpotential of H_2_ evolution on the Zn metal surface suppresses the evolution of H_2_ [46,109]. Nevertheless, in reality, the H_2_ overpotential is also influenced by various other factors, including the roughness of the Zn surface [110], operating temperature [111], and Zn concentration [112]. As a result, under certain conditions, H_2_ evolution can be observed even in mildly acidic electrolytes. Because the HER is affected by the Zn surface conditions and the interaction of the Zn anode with the electrolyte, the HER is related to the formation of dendrites. On the one hand, the dendritic growth leads to a porous structure of the Zn anode with a larger specific surface area, which provides more reaction sites for the HER. On the other hand, it can be thought that an increase in the specific surface area lowers the current density, which suppresses the HER and corrosion on the Zn surface by increasing the overpotential and forming nonconductive by-products capable of obstructing the electron transfer and Zn deposition.

Overall, the three drawbacks of Zn dendrite growth, Zn electrode corrosion, and hydrogen evolution have been discussed in the Zn metal anode. These drawbacks are not simple independent problems but are closely correlated with one another. The formation of dendrite increases the surface area of the Zn metal anode, which contributes to the accelerated hydrogen evolution. Hydrogen evolution causes a change in the local pH due to an increase in the OH^−^ concentration; however, it simultaneously accelerates the electrochemical corrosion reaction and changes the anode surface. In addition, the inert byproducts from the corrosion on the anodic surface can lead to the non-uniform surface and increased electrode polarization, which in turn facilitates the dendrite formation. Considering this complex phenomenon, it is necessary to tackle all these entangled problems with a comprehensive viewpoint rather than addressing each one of these problems separately.

## 5. Common Strategies for Modifying the Surface of Zn Metal Anodes

**Figure 10:**
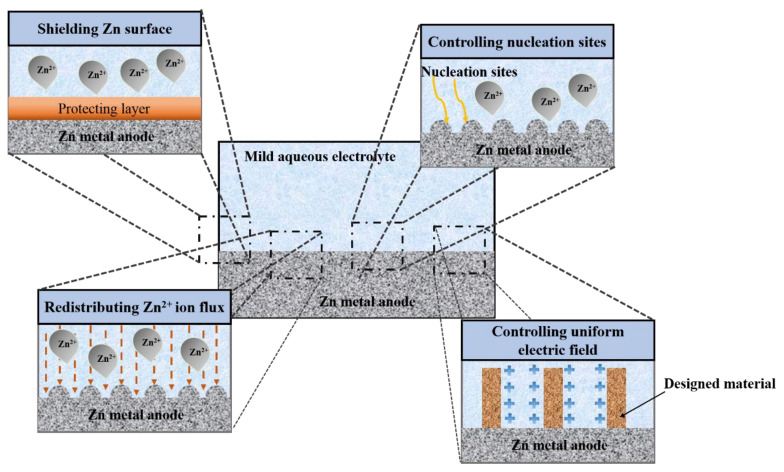
Modification strategies for enhancing the electrochemical performance of Zn metal anodes.

The electrochemical behavior of AZIBs is highly dependent on the structure of the Zn metal electrode surface. Therefore, various methods have been proposed to modify the surface of Zn metal electrodes. These methods can be categorized into several main approaches, including shielding the Zn metal to prevent side reactions, regulating the Zn deposition behavior, and creating a uniform electric field, as illustrated in Figure 10.

### 5.1. Shielding the Zn Surface

**Figure 11:**
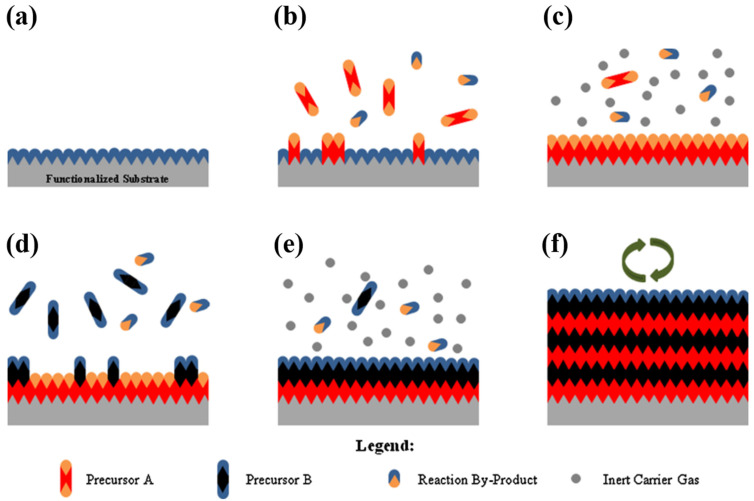
Schematic illustration of the ALD process; (**a**) the substrate surface is naturally functionalized or treated to be functionalized; (**b**) precursor A reacts with the surface after being pulsed; (**c**) an inert carrier gas is used to remove the excess precursor and by-products; (**d**) the surface reacts with the pulsed precursor B; (**e**) the inert carrier gas is used to remove excess precursor and byproducts; (**f**) repeat steps 2–5 until the desired material thickness is achieved. Reprinted with permission from Johnson et al. [114]. Copyright 2014 Elsevier Ltd.

In LIBs and other battery systems with alkali metal anodes (e.g., Na and K), the SEI is automatically formed, which acts as a protective barrier to prevent undesired reactions [45]. However, in AZIBs, an SEI is indispensable. Because most of the electrolytes used in AZIBs are highly corrosive (ZnSO_4_, ZnCl_2_, etc.) and Zn metal is quite steady in both aqueous and non-aqueous conditions, the natural formation of the SEI layer becomes insignificant. As a result, covering a Zn metal electrode with an artificial protective coating is one of the simplest yet efficient ways to enhance its stability. Preventing immediate contact between the electrode and electrolyte is the key to enhancing the electrochemical behavior of AZIBs. To overcome the harsh operational conditions of AZIBs, which are more complex than those of LIBs, the shielding material on the Zn metal anode must possess electrochemical and chemical stability. Atomic layer deposition (ALD) is an efficient technique for coating Zn metal electrodes as it offers advantages such as large coverage, conformal deposition, and precisely controllable film thickness at the nanoscale. The basic working principle of ALD relies on the chemical reactions between two or more precursors pumped alternately into a chamber containing a substrate at a specific temperature and pressure, allowing materials to be deposited in a layer-by-layer fashion on the substrate surface [113,114]. Unlike chemical vapor deposition (CVD) and other related deposition processes, ALD pumps the precursors progressively rather than simultaneously. Although ALD and CVD have certain similarities, they are different in terms of the self-limiting properties for precursor adsorption, as well as the alternate and sequential entry of the precursors and reactants [115]. A general ALD process is illustrated in Figure 11.

**Figure 12:**
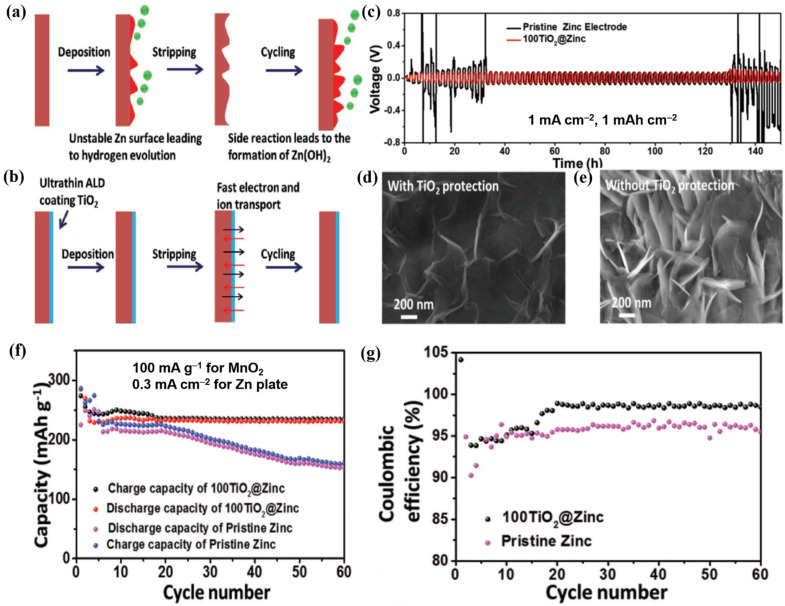
(**a**) Schematic illustration of Zn corrosion and H_2_ evolution under repeated plating/stripping cycles, (**b**) stable deposition/stripping process with a thin layer of TiO_2_ coated on the Zn anode, (**c**) symmetric cell performances of pristine Zn and TiO_2_@Zn, (**d**,**e**) ex-situ SEM images of the (**d**) TiO_2_@Zn and (**e**) pristine Zn anodes, (**f**) full cell performances of ALD TiO_2_@Zn-MnO_2_ and Zn-MnO_2_ at 100 mA g^−1^, (**g**) CEs of the ALD TiO_2_@Zn-MnO_2_ and Zn-MnO_2_ full cells at 100 mA g^−1^. Reprinted with permission from [116]. Copyright 2018 WILEY-VCH Verlag GmbH&Co. KgaA, Weinheim.

Inspired by these advantages of ALD, Zhao et al. fabricated an ultrathin TiO_2_ coating using the ALD method for the first time [116]. This passivation layer was chemically stable enough to resist the mildly acidic state and prevented the electrolyte and Zn plate electrode from coming into direct contact (as shown in Figure 12a,b). The undesirable HER was efficiently restricted under the protection of amorphous TiO_2_ (8 nm in thickness), resulting in decreased gas generation, thus reducing the risk of cell breakage by the increased internal pressure. As a result, the symmetric cell with the ALD TiO_2_@Zn electrodes had a low overpotential of 72.5 mV at 1 mA cm^−2^ and could hold it for 150 h without fluctuation, whereas the cell with the bare Zn electrodes only managed to cycle for 10 h (Figure 12c). The scanning electron microscopy (SEM) images of the electrodes cycled for 150 h revealed the presence of a large number of flakes on the surface of the Zn plate without the TiO_2_ coating (Figure 12e); however, in the case of the Zn plate coated with ALD TiO_2_, only a few flakes were visible, and their dimensions were substantially smaller than those of the flakes observed on the uncoated plate (Figure 12d). After several cycles, the hydrolysis of Zn^2+^ caused the loss of the solvent (water), leading to the formation of Zn(OH)_2_. The presence of Zn(OH)_2_ rather than Zn dendrites on the electrode surface is another issue that needs to be resolved because it is thermodynamically unfavorable in slightly acidic solutions. In this study, the full cell performance was tested using an ALD TiO_2_@Zn anode and a MnO_2_ cathode (ALD TiO_2_@Zn-MnO_2_). With TiO_2_ protection, the ALD TiO_2_@Zn-MnO_2_ full cell exhibited a discharge capacity of 235 mAh g^−1^ after 60 cycles. In contrast, the Zn–MnO_2_ cell showed a rapid capacity decay (155 mAh g^−1^ after 60 cycles) (Figure 12f). Additionally, the ALD TiO_2_ coating also enhanced the CE of the Zn plate (Figure 12g).

Inspired by Zhao’s work, He et al. [117]. coated an ultrathin Al_2_O_3_ film on a Zn metal foil via ALD. Unlike sol-gel Al_2_O_3_ coatings, the ALD Al_2_O_3_ layer was homogeneous and thin enough (10 nm) to act as a corrosion inhibitor. Because the ALD Al_2_O_3_ coating provided effective surface wetting, less electrolyte was used during the repeated Zn stripping/plating while maintaining high efficiency. The parasitic processes were successfully suppressed by shielding the Zn surface, resulting in a significant reduction of inactive by-products such as Zn(OH)_2_. Furthermore, the hydrophilic Al_2_O_3_ coating significantly improved the wettability of the Zn anode surface. It is believed that excess electrolyte plays a key role in improving the half-cell performance of Zn metal anodes. He et al. evaluated the charge-discharge profiles of symmetric cells with different electrolyte contents and found that the cells could show competitive results even at low electrolyte concentrations because of the presence of the ALD Al_2_O_3_ thin coatings. The improved wettability of the Zn foil not only reduced the charge-transfer barrier but also facilitated additional Zn^2+^ ion flux through the surface. As a result, the Al_2_O_3_@Zn symmetric cells could cycle for up to 500 h while maintaining a minimal overpotential (36.5 mV) at 1 mA cm^−2^. The Zn nucleation overpotential was related not only to the dynamics of the Zn plating/stripping reaction but also to the transfer of Zn^2+^ and electrons. Zn^2+^ and electron transport are normally the intrinsic features of the electrolyte and electrode employed; however, the Zn stripping/plating kinetics are affected by a variety of variables, including the Zn nucleus dimension and surface tension, size distribution, and nucleation substrate shape. Furthermore, the ALD Al_2_O_3_ showed a high surface wetting ability. As a result, the cells showed excellent electrochemical performance even at low electrolyte contents, which made them suitable for practical applications.

**Figure 13:**
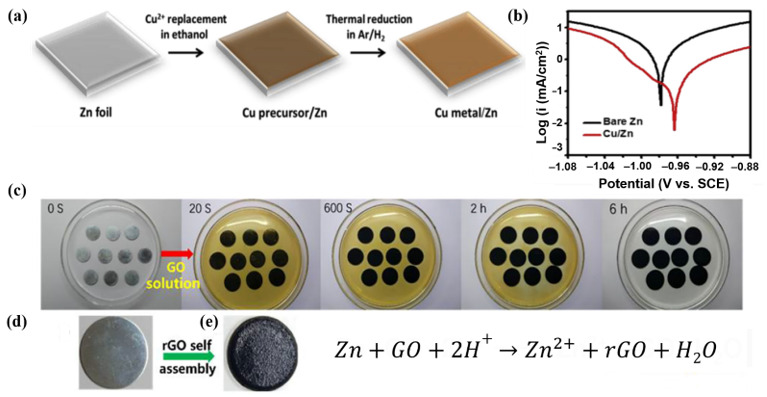
(**a**) Schematic illustration of the fabrication of the Cu-Zn electrode. (**b**) linear polarization curve of the Cu/Zn electrode in a 3 M ZnSO_4_ electrolyte. Reprinted with permission from Cai et al. [101]. Copyright 2020 Elsevier B.V. (**c**) Photographs depicting the preparation of the Zn/rGO. Zn plate (**d**) before and (**e**) after coating the rGO film. Reprinted with permission from Xia et al. [119]. Copyright 2019 Elsevier B.V.

Meanwhile, Cai et al. [101]. further explored this strategy by decorating a Zn anode with an inert metal (Cu) via a facile replacement reaction (Figure 13a). Cu shows excellent chemical stability and high conductivity in aqueous electrolytes; thus, the deposition of a Cu-rich composite surface efficiently prevents the corrosion of Zn metal. The corrosion potential of the Cu-Zn electrode (−0.964 V) was higher than that of the pristine Zn electrode (−0.976 V), which indicates that the deposition of Cu improved the chemical stability of the Zn electrode, as illustrated in Figure 13b. As compared to the pristine Zn anode, the Cu-Zn anode showed significantly high resistance. The Cu-Zn alloy (primarily Cu_5_Zn_8_) was produced in situ on the Cu/Zn anode during the electrochemical cycling, leading to the formation of a dense and Cu-rich surface layer. This alloy acted as a corrosion inhibitor and stabilizer for the electrode. Furthermore, its shape remained compact and smooth over time and prevented the deep penetration of bulk zinc metal by the electrolyte, which resulted in the formation of a significant amount of “dead Zn” and accelerated the corrosion. Another interesting approach was reported by Xie et al., who reformed a Zn plate with three-dimensional (3D)-nanoporous ZnO (3D-ZnO@Zn) [118]. The activation of the thin surface layer in the electric double layers (EDLs) by alien molecules on the inner Helmholtz plane improved the stability and long-term cycling performance of the electrode. Because ZnO had a 3D architecture with uniformly dispersed O on the surface of Zn, it not only reduced the current density by limiting the “tip effect,” but also suppressed the side-reactions at the interface and the formation of H_2_ by creating a tight exterior solvate sheath. In contrast, in the case of the bare Zn anode, Zn^2+^ showed sluggish transport kinetics into the host, resulting in strong polarization, high nucleation potential, and low stripping/plating efficiency. Another objective of this research was to gain a better understanding of the dynamic tuning of the Zn^2+^ transport toward the anode. In contrast to TiO_2_ or Al_2_O_3_, which completely shielded the Zn surface from the corrosive electrolyte, the porous ZnO layer showed strong electrostatic attraction for Zn^2+^ ions (preferably the solvated ones) in the EDL. H_2_ evolution was suppressed because of this propensity. Furthermore, because of its conductive and well-connected framework, the 3D architecture permitted quick Zn^2+^ transport, resulting in dramatically enhanced deposition kinetics. For a more precise assessment of the deposition dynamics, the exchange current density in the Zn electrodeposition process was calculated using the following equation:

(7) $$i\sim i_{0}\frac{F}{RT}\frac{\eta }{2}$$

where i is the exchange current density, i_0_ is the reference exchange current density, F is the Faraday constant, R is the gas constant, T is the absolute temperature, and η is the total overpotential. The exchange current density represents the redox reaction rate of the electrode at the equilibrium potential. The value was determined from the temperature and H_2_ concentration as well as the surface modification and cycling parameters. In addition to improving the deposition kinetics, the Arrhenius equation was used to examine the activation energy (E_a_), which can provide information on the transfer and desolvation of Zn^2+^.

(8) $$\frac{1}{R_{ct}}=Aexp\left(-\frac{E_{a}}{RT}\right)$$

where R_ct_ is the charge transfer resistance, A is the pre-exponential factor, E_a_ is the activation energy, R is the gas constant, and T is the absolute temperature. Meanwhile, Zn deposition unavoidably competed with H_2_ evolution. The HER performance of the electrode was evaluated using linear sweep voltammetry (LSV). This novel structure and artificial surface improved the Zn^2+^ deposition kinetics (nucleation potential of only 42.4 mV for 3D-ZnO@Zn vs. 66.9 mV for bare Zn; charge transfer resistance of 292.7 Ω for 3D-ZnO@Zn vs. 1240 Ω for bare Zn), reduced the de-solvation energy consumption (51.0 kJ mol^−1^ for 3D-ZnO@Zn vs. 77.2 kJ mol^−1^ for bare Zn) in the EDLs, and suppressed the HER. Besides this, 3D-ZnO@Zn showed a low current density of 7.938 mA cm^−2^, whereas pristine Zn exhibited a value of 19.68 mA cm^2^, indicating the paradoxically sluggish deposition dynamics of 3D-ZnO@Zn.

The idea of shielding the Zn surface to prevent the formation of undesired by-products is not limited to coating with an inert metal or oxide passivation layer. Xia et al. employed a casting method to modify a Zn mesh anode with reduced graphene oxide (rGO) for application in ZIBs [119]. As shown in Figure 13c, when the pretreated Zn foil was dipped into a brown aqueous solution with a dispersion of GO, the Zn surface quickly became dark, and with an increase in the incubation time, the brown GO aqueous solution turned colorless and transparent, indicating that the GO was completely reduced to rGO. Finally, the color of the Zn plate turned black (Figure 13d), indicating that a uniform black rGO layer was coated onto the Zn foil (Figure 13e). The rGO coating deposited on Zn plates offered many advantages. First, the low density of rGO increased the energy density of the full cell. Second, the layered rGO provided a flexible framework, which significantly reduced the volume change during Zn stripping/plating while improving the cyclic stability of the cell. Finally, owing to its large specific surface area, rGO promoted uniform Zn deposition during cycling and inhibited the formation of Zn dendrites on the Zn plate. In this study, the porous rGO foam on the Zn anode scaffold successfully prevented the production of Zn dendrites.

### 5.2. Regulating the Zn Deposition Behavior

#### 5.2.1. Controlling the Nucleation Sites

In traditional planar metal electrodes, uncontrollable dendrite formation has been an unresolved issue. Large protuberances on the Zn surface can pierce the separator, causing a short circuit and cell failure. The basic strategy of controlling the nucleation sites is to create additional Zn^2+^ nucleation sites, regulate the Zn plating/stripping behavior by Zn^2+^ nucleation and deposition homogeneity, restrict the dendrite growth, and prevent the Zn corrosion. As a result, several studies focusing on regulating this protrusion-forming tendency aimed to mitigate the uncontrollable dendrite formation in Zn electrodes. For instance, Cui et al. proposed the novel concept of covering Zn electrodes with Au nanoparticles (Au NPs) [120]. They decorated a Zn anode with quasi-isolated nano-Au particles to manage its Zn striping behavior, promoting the nucleation/deposition on Zn, reducing the “tip effect”, and therefore eliminating the dendritic/protuberance growth. During the stripping stage, the exposed Zn metal between the Au-NPs tended to strip faster than the Zn metal covered by the Au-NPs, leading to the formation of artificial nano-Zn tips. Because of their high Zn^2+^ affinity, the exposed Zn areas could serve as nucleation sites for directing the homogenous Zn deposition process in consecutive cycles. Because of the presence of the Au-NPs, the protrusions could be managed well and replaced by an ordered Zn plating layer even in the first few cycles. As a result, the plating layer aided the homogenous nucleation of Zn^2+^ ions, resulting in the formation of well-organized Zn flake arrays. The cell performance improved significantly because of the nucleation control mechanism, which allowed the cell to function for up to 2000 cycles with a capacity of 67 mAh g^−1^ (500 mA g^−1^).

**Figure 14:**
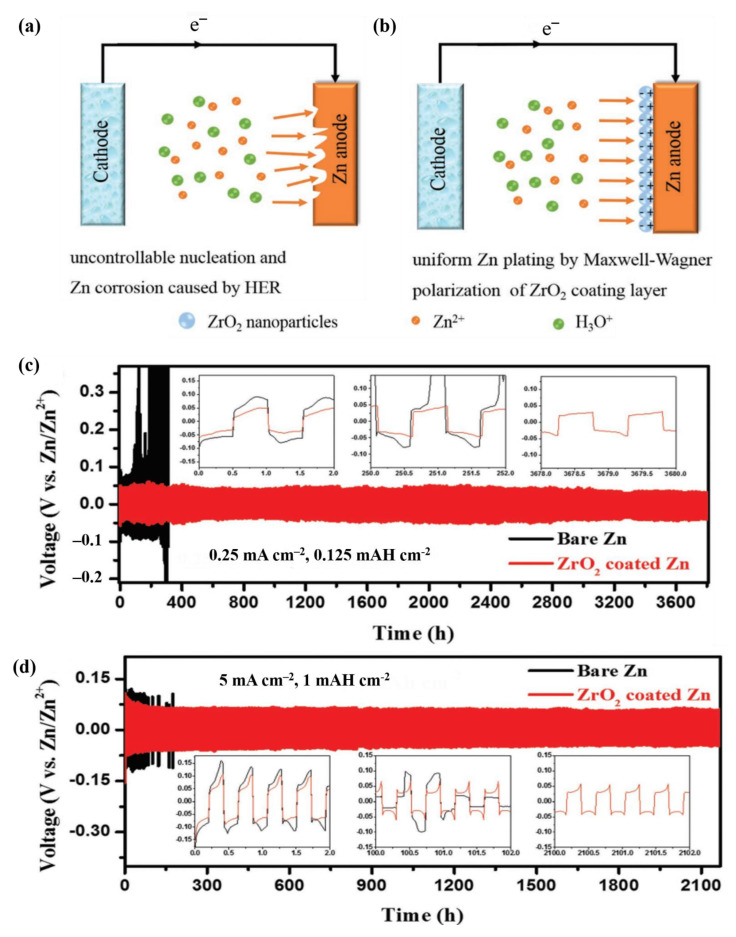
Schematics for the stripping/plating processes of (**a**) bare Zn and (**b**) ZrO_2_-coated Zn. Voltage profiles of bare Zn and ZrO_2_-coated Zn in a symmetric cell at (**c**) 0.25 mA cm^−2^ for 0.125 mAh cm^−2^ and (**d**) 5 mA cm^−2^ for 1 mAh cm^−2^. Reprinted with permission from Liang et al. [121]. Copyright 2020 WILEY-VCH Verlag GmbH&Co. KgaA, Weinheim.

Following this approach, Liang et al. exploited the Maxwell–Wagner–Sillars polarization phenomenon as the working principle for their ZrO_2_@Zn anode, which was prepared using the sol-gel method (Figure 14a,b) [121]. The Maxwell–Wagner–Sillars polarization (or more commonly, Maxwell–Wagner (MW) polarization) occurs at the interface of two structures with different relative permittivities (ε) and electrical conductivities (δ). As a result, the charges are separated over a significant distance. This phenomenon was intentionally implemented in Liang’s research by coating ZrO_2_ nanoparticles (a typical ceramic material with low electrical conductivity) onto a highly conductive Zn metal anode. Although the insulating nature of ZrO_2_ necessitated an activation step for the movement of Zn^2+^ ions through the coating layer, resulting in a high interface resistance in the first plating stage, the impedance then reduced in the subsequent cycles by the MW polarization phenomenon. The CV curves of the symmetric and complete cells with the ZrO_2_@Zn anode revealed the unexpected effects of ZrO_2_ on the stripping/plating reactions of the Zn anode. At 0.125 mAh cm^−2^, the ZrO_2_@Zn symmetric cell showed a substantially lower initial overpotential (38 mV vs. 74 mV for the bare Zn anode) and a much longer lifespan (3800 h) than the cell with the bare Zn electrode (Figure 14c). In addition, as shown in Figure 14d, the ZrO_2_-coated Zn anode showed a longer cycle lifespan (up to 2100 h) and lower polarization (32 mV) than the bare Zn anode at 5 mA cm^−2^. This can be attributed to the increase in the number of nucleation sites and the ion diffusion rate of the ZrO_2_-coated Zn anode due to the MW polarization effect. Furthermore, because ZrO_2_ is a chemically inert oxide, it can serve as a protective barrier, preventing Zn from contacting mildly acidic electrolytes. This dual-functional coating effectively reduced the uncontrollable dendrite growth and its potentially dangerous side effects. As a result, even after cycling at 5 mA cm^−2^, the ZrO_2_-coated Zn anode retained its flat and dense surface.

**Figure 15:**
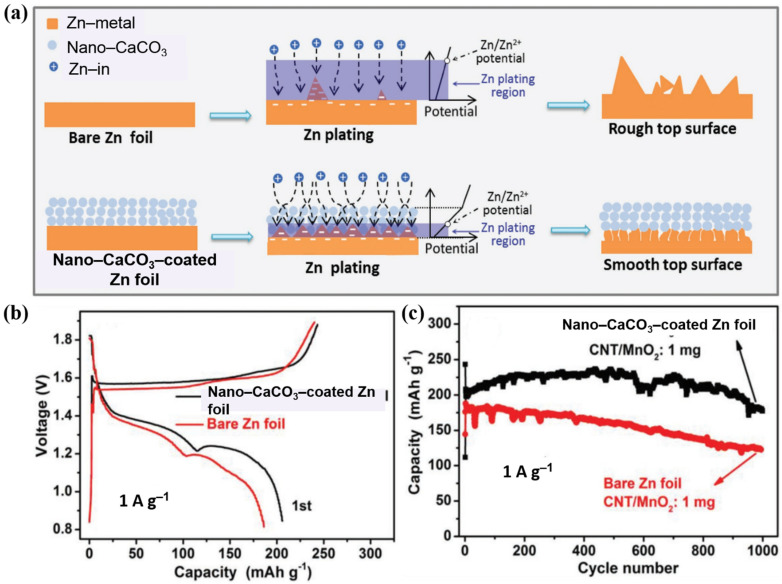
(**a**) Schematic illustration of morphology evolution for bare and nano-CaCO_3_-coated Zn foils during Zn stripping/plating cycling, (**b**) charge-discharge profiles and (**c**) full cell performance of nano-CaCO_3_-coated Zn foil and bare Zn foil. Reprinted with permission from [92]. Copyright 2018 WILEY-VCH Verlag GmbH&Co. KgaA, Weinheim.

Along this line, Kang et al. used a nanoporous CaCO_3_ coating to control the Zn deposition (Figure 15a) [92]. The holes in the CaCO_3_ coating on the Zn metal anode were crucial for controlling the formation of Zn dendrites. The electrolyte quickly penetrated the nano CaCO_3_ coating owing to its high porosity, resulting in a consistent electrolysis flux and plating of Zn throughout the Zn metal surface. After continuous running, large protuberances and detached zinc flakes were formed as a large amount of Zn was deposited in these areas. The merits of nanoporous CaCO_3_ were utilized based on this assumption. Owing to its high porosity, the nanoporous CaCO_3_ buffer layer facilitated the electrolyte flux and prevented the “local bias” behavior. In addition, the fine pores and holes at the nanoscale enclosed the Zn nuclei, thus reducing the polarization. In addition, the potential variation induced by the electrically insulating characteristics of CaCO_3_ was favorable for countering the “tip effect” as Zn^2+^ ions could be converted into Zn only in the area with enough negative potential, namely the area near the anode surface. Consequently, the battery with the nano-CaCO_3_-coated Zn anode showed a capacity of 206 mAh g^−1^ at 1 A g^−1^ and a CE of 84.7%. These values are higher than those of the pristine Zn anode (188 mAh g^−1^ and 77.5%, respectively) (Figure 15b). The battery with the nano-CaCO_3_-coated Zn anode exhibited substantially better cycling steadiness than that with the pristine Zn anode at 1 A g^−1^ (Figure 15c). Its capacity gradually increased from 206 to a maximum of 236 mAh g^−1^ in the first 500 cycles, and then reached 177 mAh g^−1^ after the 1000th cycle (capacity retention of 86%). On the other hand, the battery with the pristine Zn anode had a lower initial capacity of 188 mAh g^−1^, which remained only 124 mAh g^−1^ after 1000 cycles. Later, Zeng et al. proposed a similar strategy using conductive CNT scaffolds [122]. The highly porous CNT skeleton helped to ensure uniform seeding sites across the electrode (similar to CaCO_3_). Moreover, this flexible sheet also served as a “supplemental host” because of the occurrence of Zn stripping/plating in it, thus providing additional space for Zn deposition and alleviating the aggressive dendrite growth. More impressively, no coating process was employed; instead, the CNT scaffolds only needed to be placed between the Zn foil anode and separator, which indeed set this work apart from others because of its simplicity and scalability. In addition to applying CNTs in AZIBs, Dong et al. further applied this Zn@CNT electrode for hybrid capacitors. The Zn ion capacitor delivered remarkable stability with an average capacity of 47 mAh g^−1^ at 2 A g^−1^ (CE ~100%) for 7000 cycles.

#### 5.2.2. Redistributing Zn^2+^ Ion Flux

Another efficient strategy to improve the Zn deposition behavior of Zn metal anodes is to redistribute the Zn^2+^ ion flux. As a pioneer in this field, Zhao et al. proposed a solid-state interphase comprising of polyamide (PA) and zinc trifluoromethane sulfonate (Zn(TfO)_2_) [94]. They demonstrated that ions tended to move horizontally across the electrode surface and aggregated at already existing nucleation sites to optimize the surface energy. However, this inclination was intentionally restrained by introducing the PA chains. Because of their higher energy barrier to diffuse laterally, the Zn^2+^ ions were obligated to deposit at the premier position where they were initially adsorbed through the interface, resulting in an increase in the number of nucleation sites. In addition, the synergistic interaction between the Zn^2+^ ions and polar groups (C=O) in the PA backbones improved the nucleation overpotential, which favored the formation of smaller nuclei. These dual effects ultimately led to the formation of a dense and smooth Zn layer when the cells were continuously cycled. It is important to note that the PA coating did not allow electrons to cross over it, preventing the external reduction of Zn^2+^. Therefore, the electrolyte could not contact the newly formed Zn without shielding. The water-resistant characteristics of the coating also suppressed the adverse HERs. The carbonyl-rich networks offered plentiful hydrogen bonds to bind and impair the Zn^2+^ ion solvation-sheath, preventing H_2_O and O_2_ from participating in such reactions. As a result, the discharge capacity of the Zn/MnO_2_ battery increased with an increase in the number of cycles from 450 (below 30 mAh g^−1^) to 1000 (155.4 mAh g^−1^, 88% capacity retention) at 2C.

**Figure 16:**
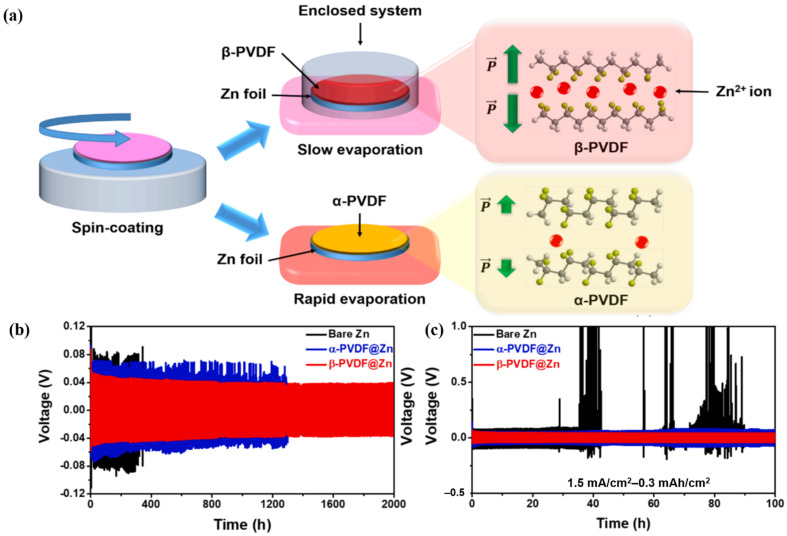
(**a**) Schematic illustration of the β- and α-PVDF coating processes, (**b**) long-term profiles of β-PVDF@Zn (red), α-PVDF@Zn (blue), and bare Zn (black) with symmetrical cells at a current density of (**b**) 0.25–0.05 mAh cm^−2^, (**c**) 1.5–0.3 mAh cm^−2^. Reprinted with permission from Hieu et al. [123]. Copyright 2021 Elsevier B.V.

Although the coating of metal anodes with polymers has been widely practiced with positive results, an appropriate polymer should be chosen based on some fundamental principles. To withstand the volume changes during repeated cycling, a metal anode must be mechanically strong and flexible. Chemical stability, water insolubility, and hydrophilicity are also essential features of an ideal metal anode. Finally, the polymer network used for coating Zn anodes must possess abundant polar groups to interact with the metal ions. Considering these requirements, polyvinylidene fluoride (PVDF) has been used to modify the surface of Zn anodes via a facile spin-coating process (Figure 16a) [123]. Under slow evaporation conditions, the F atoms in the PVDF side chains rearrange to form an all-trans conformation known as the β-phase, in which all the dipoles align on the same side. Owing to its high polarity, β-PVDF shows excellent ferroelectric, piezoelectric, and pyroelectric properties. When symmetric β-PVDF@Zn|ZnSO_4_|β-PVDF@Zn was cycled at 0.25 mA cm^−2^ and an area capacity of 0.05 mAh cm^−2^, a small overpotential was achieved even after 2000 h of operation. This performance is far superior to that shown by Zn|ZnSO_4_|Zn, which withered after 200 h (Figure 16b). This superior performance of β-PVDF@Zn|ZnSO_4_|β-PVDF@Zn can be attributed to the presence of the multifunctional β-PVDF membrane in it. On the one hand, the highly electronegative C-F alignments acted as preferable diffusion paths for Zn^2+^ and distributed these ions uniformly onto the entire anode surface. This efficient channel network helped to abate the local current density and developed a homogeneous layer of Zn plating. On the other hand, owing to its resilience, the β-PVDF polymer could withstand dendrite growth and hindered the formation of inactive side-products (ZnO) in acidic electrolytes. Protected by the versatile β-PVDF cover, the Zn anode retained its morphology without serious damage and, as a result, the full cell delivered an appealing cycling performance (discharge capacity of 60 mAh g^−1^ after 4000 cycles).

With regard to PVDF, Liu et al. synthesized a MOF-PVDF composite coating layer consisting of PVDF and hydrophilic microporous metal-organic framework (MOF) particles [124]. The metal ions could evenly access the anode surface because of their low wettability with common aqueous electrolytes, resulting in local ion deposition and troublesome protrusion growth. The microporous architecture of the MOF induced a wetting effect on the Zn electrode at the nanoscale, generating a hydrophilic surface. Each MOF nanoparticle made intimate contact with Zn and served as a nanoscale electrolyte reservoir. As a result, the ion flux cooperated well with the Zn plate to the greatest extent, promoting a uniform Zn stripping/plating process. Furthermore, the compact MOF particles enabled fast ion diffusion through the fully wetted surface, dramatically lowering the charge-transfer resistance.

### 5.3. Creating Uniform Electric Field

**Figure 17:**
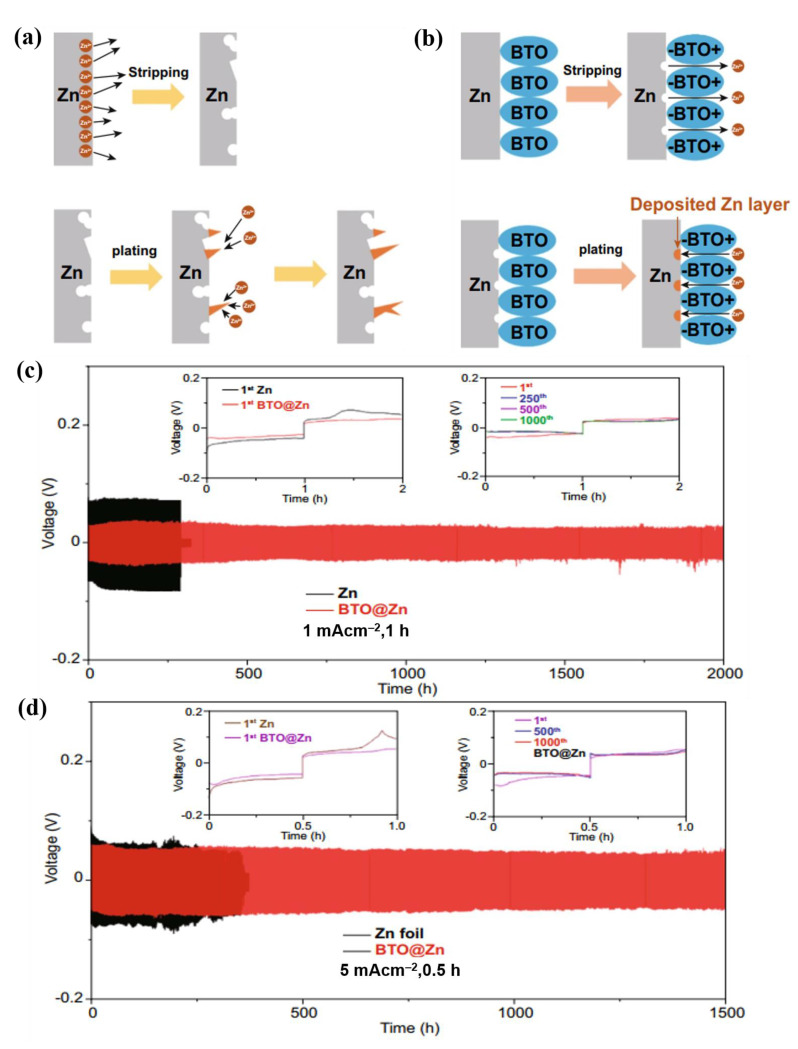
Schematic of Zn ion transport during Zn stripping/plating for (**a**) bare Zn, and (**b**) BTO@ Zn foil. Cyclic performances of the symmetric cells with Zn and BTO@Zn at (**c**) 1 mA cm^−2^ (1 mAh cm^−2^), and (**d**) 5 mA cm^−2^ (2.5 mAh cm^−2^). Reprinted with permission from [125].

One of the most important issues affecting the stability of Zn anodes is the formation of Zn dendrites. Engineering a synthetic SEI on the surface of Zn is an effective method for limiting the Zn dendrite formation. The fundamental goal of this strategy is to develop materials that can direct the orderly migration of Zn ions while providing a homogenous electric field at the electrode-electrolyte interface, preventing the formation of Zn dendrites. Recently, it has been demonstrated that an artificial SEI based on BaTiO_3_ (BTO) can effectively restrain the Zn dendrite growth (Figure 17a,b) [125]. The polarized BTO layer played a significant role in controlling the orderly migration of Zn ions because of the uniform electric field through it. The polarization of BTO was induced by the Ti ions that deviated from the center of the symmetrical site in BTO under an external electric field. In this situation, the two-dimensional diffusion of Zn ions was highly restricted, and the vertical movement of Zn ions through the BTO layer was thermodynamically preferable. Moreover, according to the density functional theory calculation results, the formation energy of Zn deposition was much lower for BTO@Zn than that for bare Zn (−2.28 vs. −0.35 eV), thus BTO promoted dense Zn plating without dendrites. In addition, the water molecules in the solvated Zn ions (Zn(H_2_O)_6_^2+^) were easily attracted by the O atom in BTO via hydrogen bonding, which facilitated the diffusion of Zn ions and restricted the Zn dendrite formation [91]. In contrast, in the absence of the BTO coating, the Zn dendrite formation could not be regulated because of the uneven deposition of Zn ions as well as the preferential deposition at the protruded surfaces. As a consequence, the BTO@Zn-symmetric cell showed cycling stability for more than 2000 h (1000 cycles) at 1 mA cm^−2^, 1 mAh cm^−2^ (Figure 17c) and for 1500 cycles at 5 mA cm^−2^, 2.5 mAh cm^−2^ (Figure 17d). After 300 cycles at 2 A g^−1^, the BTO@Zn–MnO_2_ full cell battery showed high rate capability and approximately 100% CE in a mild aqueous electrolyte.

**Figure 18:**
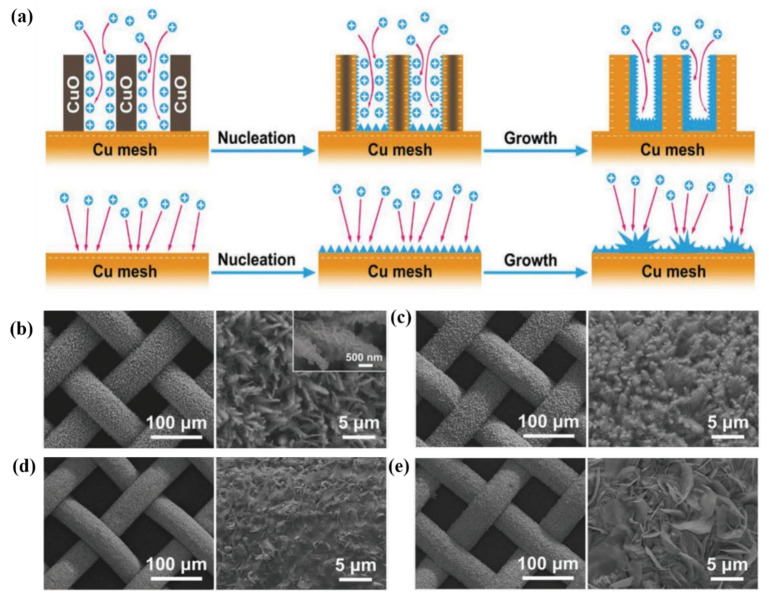
(**a**) Schematic illustration of Zn deposition on CM@CuO and CM. SEM images of CM@CuO@Zn with the capacities of (**b**) 1 and (**c**) 5 mAh cm^−2^. SEM images of CM@Zn with the capacities of (**d**) 1 and (**e**) 5 mAh cm^−2^. Reprinted with permission from [126]. Copyright 2020 Wiley-VCH GmbH.

Zhang et al. investigated CuO nanowires grown on a Cu mesh (CM@CuO) by anodic oxidation, followed by thermal annealing in air [126]. Because of the decreased energy barrier and increased number of active sites, Zn^2+^ was preferentially absorbed by CuO and dispersed evenly on the Zn surface, which was advantageous for Zn nucleation. Furthermore, as illustrated in Figure 18a, the Cu generated from CuO enhanced the electrical conductance, which in turn increased the electric field during the Zn nucleation, resulting in a more ordered Zn growth. Because of the increased zincophilicity and uniform Zn^2+^ distribution on the surface, small Zn nanosheets were consistently developed on the CuO nanowires, as shown in the SEM image (Figure 18b). Zn remained within the CuO nanowire structure even when the capacity was increased to 5 mAh cm^−2^ (Figure 18c). In contrast to the dendrite-free plating behavior of CM@CuO@Zn, the early Zn growth on the pristine CM was uneven, and micron-sized Zn dendrites (5 µm) were observed in the CM@Zn anode (Figure 18d,e). This work demonstrated that CM@CuO@Zn shows excellent electrochemical performance owing to its uniform electric field and 3D structure. The as-prepared Zn anode showed excellent cycling stability (340 h) and low voltage hysteresis (20 mV) in symmetric cells at 1 mA cm^−2^ and 1 mAh cm^−2^. Meanwhile, a 3D hierarchical N-doped carbon cloth (NC) was produced using magnetron sputtering as a scaffold combining a 3D architecture and an interfacial control to efficiently promote the uniform nucleation of Zn metal and reduce the growth of Zn dendrites [127]. The dynamics of Zn^2+^ transfer and deposition could be accelerated by the desolvation process of Zn^2+^ with surface chemistry control. The 3D hierarchical conductive carbon scaffold uniformly distributed the electric fields and lowered the local current density. The N-containing functional groups in NC served as nucleation sites to distribute the Zn nuclei uniformly across the electrode surface. As Zn was constantly deposited, an electric charge was generated in the uneven nucleation sites. The carbon scaffold doped with N atoms encouraged the homogenous nucleation and growth of Zn, thus constraining the formation of Zn dendrites. The N atoms generated by the addition of pyrrole lowered the migration energy barrier and promoted the redistribution of Zn ions. Furthermore, the zincophilicity of the NC scaffold was enhanced by the strong electrostatic interaction between the negatively charged pyrrole N sites in the carbon lattice and the Zn atoms, resulting in the homogeneous nucleation and growth of Zn. The negative state of the pyrrole N site can be explained by the formation of σ-bonds between the doped N atoms and the adjacent carbons from which electrons moved toward pyrrole N through the inductive effect. As a result, the assembled half-cell showed a high zinc stripping/plating CE of 98.8%. The NC-Zn symmetrical cell showed stable operation at a high current density of 5 mA cm^−2^ with an overpotential as low as 11 mV after 210 cycles.

The various modification principles used for modifying Zn anodes and their performances (symmetric and full cell) for AZIBs in mildly acidic electrolytes are summarized in Table 2.

According to the strategies discussed above for modifying the Zn metal surface, the drawbacks must be addressed concurrently to achieve a practical improvement of Zn metal anodes. As for the dendrites, they can be effectively suppressed by increasing the Zn nucleation sites, redistributing the ionic flux of Zn^2+^, and controlling the electric field. Corrosion and HER can be resolved by reducing the chemical activity of H_2_O and introducing the protective layer on the Zn metal anode. As can be seen from Table 2, these strategies improve the performance of the Zn anodes compared to the pristine state, but some of them are not still viable enough for practical applications in the perspectives of overall performance. For example, the use of carbon-based materials with a high specific surface area such as rGO can inhibit the dendrite formation, but can accelerate the corrosion, which may result in the degraded full-cell performance. Therefore, the effectiveness of modification methods should be strictly evaluated considering various performance metrics together to realize the high-performance Zn anode.

## 6. Conclusions and Outlook

In summary, the major challenges associated with the application of Zn metal anodes in mild aqueous electrolytes and the modification strategies employed to overcome these challenges are discussed to provide an insight into the development of high-performance AZIBs. AZIBs are regarded as promising alternatives to LIBs (especially for large-scale energy storage systems) owing to their safety, cost-effectiveness, environmental benignity, and high energy density. The main challenges associated with the use of Zn metal anodes in neutral or mildly acidic media are dendrite growth, corrosion, and H_2_ evolution. The presence of uneven nucleation sites on Zn metal facilitates uncontrollable charge transfer, which accelerates the generation of an uneven electric field, causing the growth of dendrites. The corrosion of Zn metal anodes can be categorized as self-corrosion or corrosion by an electrochemical reaction. The latter refers to the irreversible Zn consumption due to the removal of Zn from the electrode as well as the side reactions between the electrode and electrolyte. H_2_ evolution is a complicated process that is affected by the reduction potential, overpotential, surface area, electrolyte pH, and operating temperature. To address these issues, researchers have proposed various modification strategies, which can largely be categorized as (i) shielding the Zn metal from side reactions, (ii) regulating the Zn deposition behavior (controlling the nucleation sites and redistributing the Zn^2+^ ion flux), and (iii) establishing a uniform electric field.

Despite the considerable progress made in this field, there is still a long way to go to address the complex challenges discussed thus far. An efficient approach to produce a dendrite-free and highly reversible Zn anode is to strategically tailor the 3D structure of the electrode. The construction of 3D nanostructured Zn metal anodes has not been fully explored yet. Fabricating Zn anodes with hierarchical 3D architectures provides new opportunities for increasing the rate capability and service life of AZIBs because it allows a more uniform distribution of the electric current and provides a large number of reaction sites. The fabrication of 3D Zn architecture can be realized by the coating of Zn metal (e.g., electrodeposition) on a well-defined conductive 3D framework (e.g., metal form, porous carbon, carbon mesh, etc.) or by the selective etching of the sacrificial components in Zn composites. The caveat here is that while the increase in the surface area of the electrode can improve the energy density and suppress the dendrite development, it can accelerate the corrosion of Zn metal. This can be avoided by coating 3D Zn metal with corrosion inhibitors or by introducing organic/inorganic additives in the plating electrolyte. In addition, although the behavior of Zn electrodes in mild aqueous electrolytes differs from that in alkaline electrolytes, the knowledge of the operation of Zn batteries in alkaline aqueous media can provide time-saving guidance for the study of AZIBs. Furthermore, more progress can be made by investigating the previously reported strategies for modifying metal anodes (e.g., in Li/Na metal batteries).

In addition to the Zn metal anode, the other components (cathode and electrolyte) of the cell should also be taken into consideration to improve the performance of the anode. The type of electrolyte, electrolyte concentration, and additives are important factors that strongly influence the electrochemical reactions on the Zn metal anode. In particular, in mild aqueous electrolytes, ZnSO_4_ and Zn(CF_3_SO_3_)_2_ are mainly used within a certain concentration range. In most cases, ZnSO_4_ is preferable because of its relatively low price as compared to that of Zn(CF_3_SO_3_)_2_. However, ZnSO_4_ suffers from the formation of hydroxide sulfate by-products (Zn_4_(OH)_6_SO_4_·nH_2_O). Exploring other Zn salts for application under mild aqueous conditions and some appropriate ZnSO_4_-based additives is another potential approach. Mn-based materials are the most widely used cathode materials. As revealed by many studies on AZIBs cathodes, Mn-based oxides can be operated at high working voltages, but it is still difficult to fully understand their reaction mechanism because of the diverse phase transitions occurring during the reactions. Apart from Mn-based cathode materials, V-based materials and Prussian blue analogs can be potential candidates for application in conjunction with Zn metal anodes for AZIBs. However, in this case, the limitations of these materials (V-based oxides: low operation voltage, Prussian blue analogs: low specific capacity) should be considered.

Finally, most studies on Zn metal anodes have mainly focused on coin cells or pouch cells. To meet the market demands (especially in large-scale energy storage), these studies need to be expanded to larger cells (e.g., cylindrical and prismatic cells). In practical applications, unknown challenges that have not been encountered in coin cells can be encountered. The know-how of various cell configurations can narrow the gap between the lab-scale results and the commercialization of AZIBs. Finally, the development of flexible and wearable AZIBs can be an intriguing research topic because of their intrinsic safety, low cost, and facile fabrication (without the need for a glovebox). To realize this, not only should a sufficiently thin Zn metal electrode (beyond the commercial Zn foil) be developed, but appropriate components (cathode, solid electrolyte, and packing material) compatible with Zn anodes should also be carefully designed.

## Figures and Tables

**Table 1:** List of components used in the typical coin-cell assembly of the ZIB with Zn metal anode [82].

Components	Representative Material
Anode material	Zn foil (80.0 mm in diameter, 0.25 mm in thickness)
Cathode material	α-MnO_2_, β-MnO_2_, V-based materials, Prussian blue analogues
Cathode current collector	Stainless steel spring (15.4 mm in diameter and 1.1 mm in thickness)
Separator	Whatman glass fiber filter
Electrolyte	2 M ZnSO_4_ with 0.1 M MnSO_4_

**Table 2:** Modification mechanisms and electrochemical performances of recently reported Zn metal anodes in mildly acidic electrolytes for AZIBs.

Anode	Mechanism	Corrosion Potential (V) (vs. Ag/AgCl)	Symmetrical Cell Performance:Lifespan (h), Capacity, and Current Density	Full-cell Performance:Capacity (mAh g^−1^)/Cycle/Capacity Retention (%)/Current Density (mA g^−1^)	Ref
TiO_2_@Zn	Shielding Zn surface	−0.89	150 h 1 mA cm^−2^, 1 mAhcm^−2^	134 mAh g^−1^/1000 cycles/85%/1 A g^−1^	[116]
Al_2_O_3_@Zn	Shielding Zn surface	−0.88	500 h 1 mA cm^−2^, 1 mAh cm^−2^	158 mAh g^−1^/1000 cycles/89%/1 A g^−1^	[117]
Cu-Zn/Zn	Shielding Zn surface	−0.96	1500 h 1 mA cm^−2^, 0.5 mAh cm^−2^	-	[101]
rGO@Zn	Shielding Zn surface	-	300 h 1 mA cm^−2^, 0.5 mAh cm^−2^	61 mAh g^−1^/5000 cycles/86%/1 A g^−1^	[119]
NA-Zn	Controlling nucleation sites	-	2000 h 0.25 mA cm^−2^, 0.05 mAh cm^−2^	67 mAh g^−1^/2000 cycles/-/0.5 A g^−1^	[120]
ZrO_2_@ Zn	Controlling nucleation sites	-	2100 h 5 mA cm^−2^, 1 mAh cm^−2^	52 mAh g^−1^/3000 cycles/42%/1 A g^−1^	[121]
CaCO3@Zn	Controlling nucleation sites	-	900 h 0.25 mA cm^−2^, 0.05 mAh cm^−2^	177 mAh g^−1^/1000 cycles/86%/1 A g^−1^	[92]
CNT@Zn	Controlling nucleation sites	-	200 h 2 mA cm^−2^, 2 mAh cm^−2^	167 mAh g^−1^/1000 cycles/89%/-	[122]
PA@Zn	Redistributing Zn^2+^ ion flux	−0.96	8000 h 0.5 mA cm^−2^, 0.25 mAh cm^−2^	154 mAh g^−1^/1000 cycles/88%/0.6 A g^−1^	[94]
PVDF@Zn	Redistributing Zn^2+^ ion flux	-	2000 h 0.25 mA cm^−2^, 0.05 mAh cm^−2^	57 mAh g^−1^/2000 cycles/-/1 A g^−1^	[123]
MOF-PVDF@Zn	Redistributing Zn^2+^ ion flux	-	500 h 1 mA cm^−2^, 0.5 mAh cm^−2^	-	[124]
BTO@Zn	Creating uniform electric field	-	1500 h 5 mA cm^−2^, 0.5 mAh cm^−2^	74 mAh g^−1^/300 cycles/67%/2 A g^−1^	[125]
CM@CuO@Zn	Creating uniform electric field	-	350 h 1 mA cm^−2^, 1 mAh cm^−2^	-	[126]
NC@Zn	Creating uniform electric field	-	300 h 1 mA cm^−2^, 0.5 mAh cm^−2^	-	[127]

## Data Availability

Not applicable.
